# New semisynthetic α-glucosidase inhibitor from a doubly-chemically engineered extract

**DOI:** 10.1007/s13659-024-00488-2

**Published:** 2025-01-05

**Authors:** María I. Osella, Mario O. Salazar, Carlos M. Solís, Ricardo L. E. Furlan

**Affiliations:** 1 Consejo Nacional de Investigaciones Científicas y Técnicas, Suipacha 531, S2002LRK Rosario, Argentina; 2 Farmacognosia, Facultad de Ciencias Bioquímicas y Farmacéuticas, Universidad Nacional de Rosario, Suipacha 531, S2002LRK Rosario, Argentina

**Keywords:** Natural products, Chemically engineered extracts, Glucosidase inhibitor, Pyrazole, Fluorine

## Abstract

Chemically engineered extracts represent a promising source of new bioactive semi-synthetic molecules. Prepared through direct derivatization of natural extracts, they can include constituents enriched with elements and sub-structures that are less common in natural products compared to drugs. Fourteen such extracts were prepared through sequential reactions with hydrazine and a fluorinating reagent, and their α-glucosidase inhibition properties were compared. For the most bioactive mixture, a chemically modified propolis extract, enzyme inhibition increased 22 times due to the reaction sequence. Bio-guided fractionation led to the isolation of a new fluorinated pyrazole produced within the extract by chemical transformation of the flavonoid chrysin. The inhibitor results from the action of the two reagents used on four common functional groups present in natural products (carbonyl, phenol, aromatic carbon, and a double bond). The reactions led to the opening of a 6-member oxygenated heterocycle to produce a 5-member nitrogenated one, as well as the dehydroxylation and fluorination in two different positions of one of the aromatic rings of the natural starting material, all within a complex mixture of natural products. Overall, these transformations led to an approximately 20-fold increase in the α-glucosidase inhibition by the isolated inhibitor compared to its natural precursor.

**Graphical Abstract:**

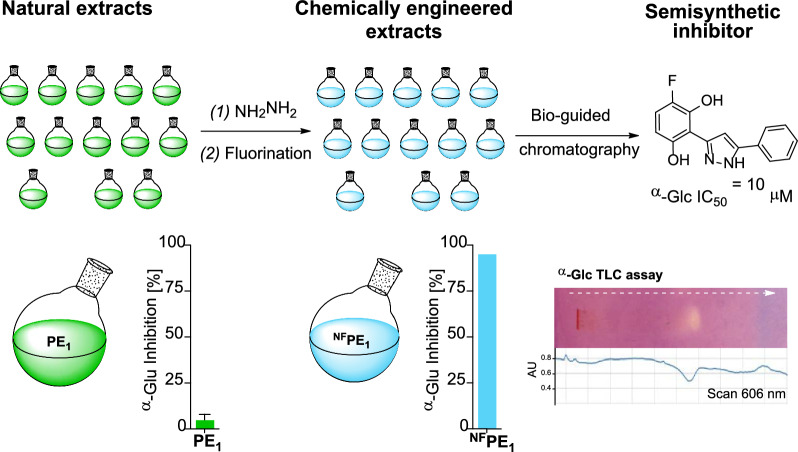

**Supplementary Information:**

The online version contains supplementary material available at 10.1007/s13659-024-00488-2.

## Introduction

The chemical engineering of natural product extracts can lead to semisynthetic compound mixtures with expanded diversity and improved biological properties [1–5]. Depending on the reaction protocol, the components of these chemically engineered extracts can be enriched in different halogens [6–9] or heteroatoms [10–18], and/or skeletons that are different from those of their natural precursors [1, 10, 11, 19–23]. Two reactions that have been successfully applied individually to modify natural mixtures are those with hydrazine [5] and with Selectfluor [7]. However, their sequential combination has not yet been explored.

Chemically engineered extracts (CEEs) have yielded various bioactive compounds, such as cytotoxic, [20, 23] antimicrobial, [6, 24, 25] and antiparasitic agents, [10, 26] as well as DNA-binding molecules [27] and enzyme inhibitors [7–9, 28], including some glucosidase inhibitors [11–13].

In humans, α-glucosidase (α-Glc) is a key digestive enzyme that catalyzes the hydrolytic cleavage of disaccharides (maltose and sucrose) into monosaccharides (glucose and fructose) in the intestine. Hence, the inhibition of α-Glc activity can retard the elevation of blood sugar, suppressing postprandial hyperglycemia, [29] and represents a useful strategy to control postprandial glucose level imbalance in type 2 diabetes patients [30]. Diabetes mellitus is among the top ten diseases in terms of impact on global health, [31] currently affecting approximately 9% of the adult population aged 20–79 years (425 million) [32]. Due to deficient insulin production (type 1 diabetes) or use (type 2 diabetes), patients experience abnormally high blood glucose levels which, if prolonged, can lead to severe cardiac, renal, cerebral, vascular, and visual complications [33–35].

Essential oils (EOs) are complex mixtures of natural hydrophobic small molecules derived from plant secondary metabolism [36, 37]. In nature, its production is diversity-oriented and regulates the interaction with other living organisms (plants, insects, or animals). EOs are increasingly recognized as valuable sources of molecules for drug discovery [37], exhibiting a wide range of biological activities [36]. There are only a few examples of chemically engineered essential oils, produced by halogenation [7, 8], sulfonylation [15], or thiocyanation [38], from which xanthine oxidase, tyrosinase, acetylcholinesterase, and glucosidase inhibitors have been identified.

Propolis are natural resinous mixtures produced by honeybees from substances collected from tree buds, exudates, and other botanical materials. Over the last two decades, they have gained significant scientific and commercial interest due to their broad clinical applications [39–41]. The chemical composition of propolis is highly complex, with over 500 compounds identified thus far, predominantly including terpenoids and phenolics (such as phenylpropanoids, flavonoids, lignans, coumarins, xanthones, etc.) [42, 43]. There are no reports on preparing chemically engineered propolis extracts (PEs).

The objective of this study was to explore whether the application of two sequential chemical reactions could lead to the identification of a new α-Glc inhibitor from the resulting chemically engineered extract. This investigation involved the chemical modification of 14 natural mixtures (essential oils and propolis extracts) through sequential reactions with hydrazine and Selectfluor® to introduce nitrogen and fluorine atoms into the natural molecular skeletons. While these reagents have been used individually in the past to create CEEs, their combined, sequential use is investigated here for the first time. Some of the resulting CEEs inhibited the enzyme α-Glc, and a fluorinated pyrazole was identified in the most active mixture. This compound, generated from the flavonoid chrysin within the extract, inhibits α-Glc more effectively than acarbose.

## Results and discussion

### Chemical diversification of natural mixtures and impact on α-Glc inhibitory properties

**Fig. 1:**
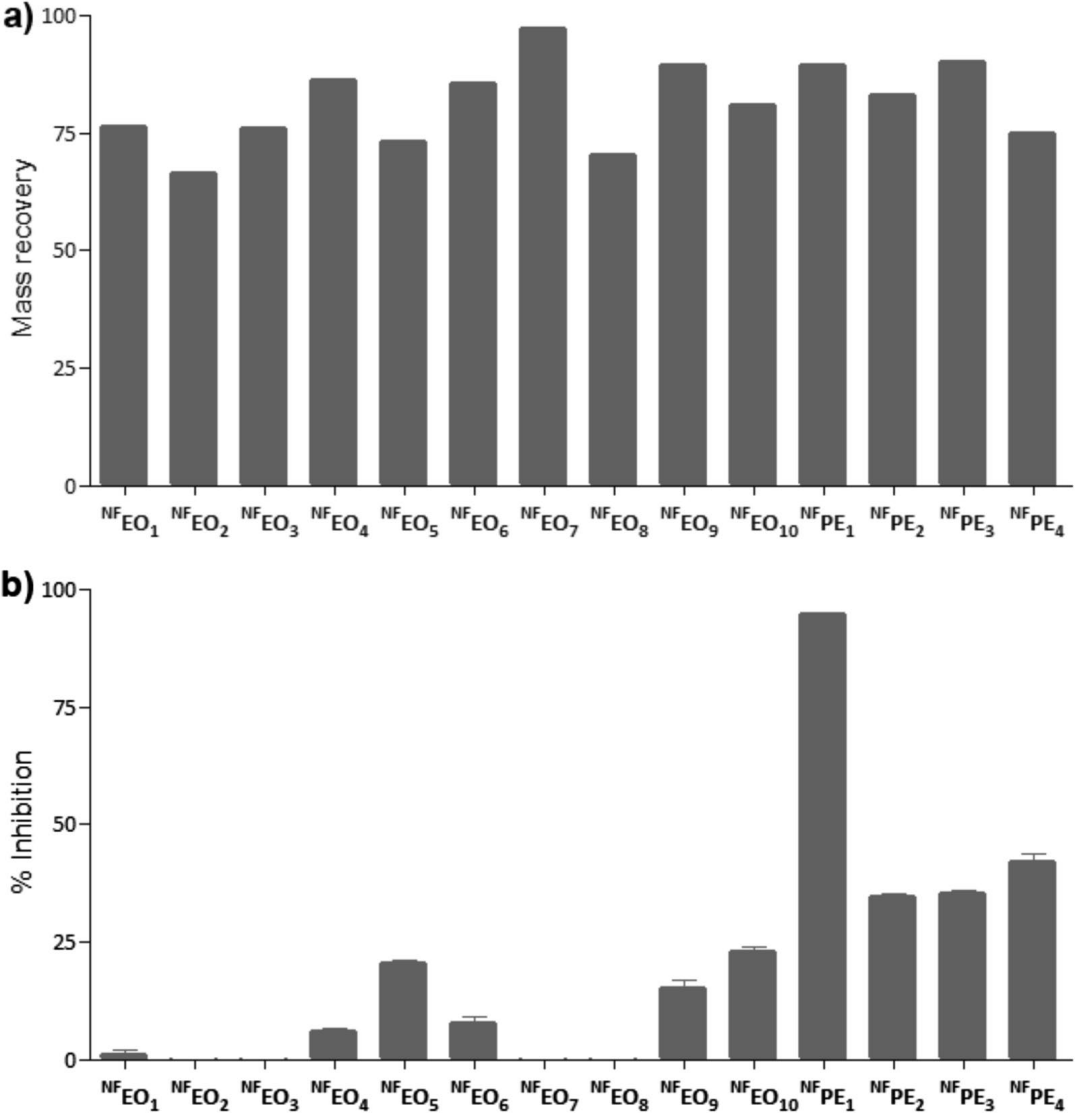
**a** Mass recovery and **b** α-Glc inhibition at 3.125 µg/mL for ^NF^EO_1_ (*S. officinalis*), ^NF^EO_2_ (*J. communis*), ^NF^EO_3_ (*J. virginiana*), ^NF^EO_4_ (*C. cassia*), ^NF^EO_5_ (*T. occidentalis*), ^NF^EO_6_ (*L. cubeba*), ^NF^EO_7_ (*C. citratus*), ^NF^EO_8_ (*A. absinthium*), ^NF^EO_9_ (*E. caryophyllata*), ^NF^EO_10_ (*C. odorata*), ^NF^PE_1_ (Esperanza, Santa Fe province), ^NF^PE_2_ (Reconquista, Santa Fe province, PE2), ^NF^PE_3_ (Lucas González, Entre Ríos province), and ^NF^PE_4_ (Dean Funes, Córdoba province)

14 natural mixtures, 10 EOs and 4 PEs, were subjected to a reaction with hydrazine and, after removing the excess reagent, with Selectfluor®. The mass recovery for EOs ranged from 62 to 105% (average 94%) for the first reaction, 66% to 97% for the second reaction (average 80%), and 48 to 89% for the entire sequential process (average 70%) (Fig. 1a and Table S1). For PEs, the mass recovery ranged from 52 to 83% (average 67%) after the first reaction, 75% to 90% for the second reaction (average 84%), and 42% to 75% for the entire sequential process (average 57%) (Fig. 1a and Table S1).

To evaluate their potential as a source of α-Glc inhibitors, the inhibition properties of the series of doubly chemically modified extracts were compared. When tested at 3.125 µg/mL, the CEEs from EOs exhibited enzyme inhibitions below 25%, whereas the CEEs from propolis showed inhibitions between 34 and 95% (Fig. 1b). The most interesting CEE of the series was the doubly modified extract from propolis sample number one (^**NF**^**PE**_**1**_) that displayed 95% inhibition (and close to 50% inhibition in a 1/16 dilution (0.781 µg/mL) (Fig. S1).

**Fig. 2:**
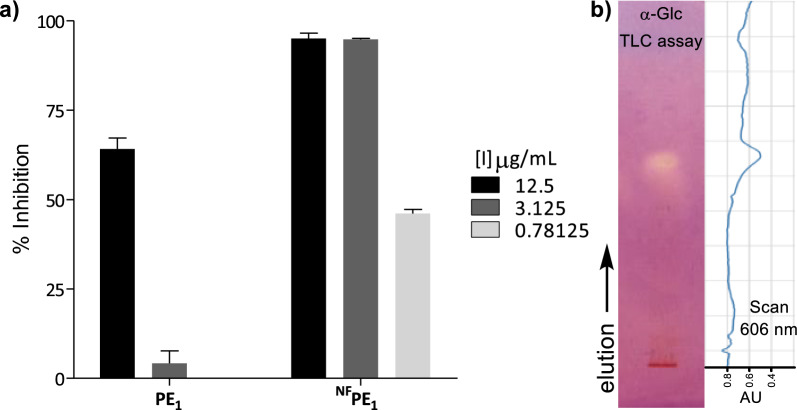
**a** α-Glc inhibition at 12.5, 3.125, and 0.781 µg/mL by PE_1_ *vs.* ^NF^PE_1_ **b** TLC of ^NF^PE_1_ revealed by α-Glc inhibition and scan of the TLC-plate at 606 nm (absorption wavelength of the diazonium dye). Silica gel, hexane–ethyl acetate, 1:1, *V/V*

When compared with its precursor propolis extract (**PE**_**1**_), the chemically engineered ^**NF**^**PE**_**1**_ produced significantly better inhibition at the three concentrations tested (12.5, 3.125, and 0.781 µg/mL) (Fig. 2a). This increased α-Glc inhibitory activity could be due to the presence of new semisynthetic compounds resulting from the incorporation of fluorine and/or nitrogen.

It is worth mentioning that, since ^**NF**^**PE**_**1**_ is a compound mixture of unknown composition, the observed α-Glc inhibition could result from the action of one good inhibitor, many weak inhibitors, or any other intermediate situation. These different scenarios define the real potential of a bioactive semisynthetic library as a source of hit compounds, so having this information beforehand facilitates early decision-making. Therefore, the α-Glc inhibition by ^**NF**^**PE**_**1**_ was analyzed by effect-directed analysis on thin-layer chromatography (TLC), a technique particularly suited to assess the inhibition properties of compound mixtures [44–46].

This format assay allows measuring the inhibitory properties of a sample chromatographed on a thin layer, by covering it with a gel containing enzyme, substrate, and a revealing reagent for the product. For α-Glc, the TLC assay relies on the enzymatic hydrolysis of the substrate 2-naphthyl-α-D-glucopyranoside to form 1-naphthol, which reacts with Fast Blue B salt to produce a purple-colored diazonium dye [47]. Therefore, regions of the chromatogram containing α-Glc inhibitors appear as clear spots against the purple background and can be observed directly or by scanning the TLC at the dye absorption wavelength (606 nm). ^**NF**^**PE**_**1**_ exhibited only one spot on the TLC-bioassay (Fig. 2b), suggesting that the inhibition observed for this mixture is due to one compound or a few compounds with similar chromatographic behavior.

### Identification of an α-Glc inhibitor from the propolis doubly chemically modified extract ^NF^PE_1_

**Scheme 1:**
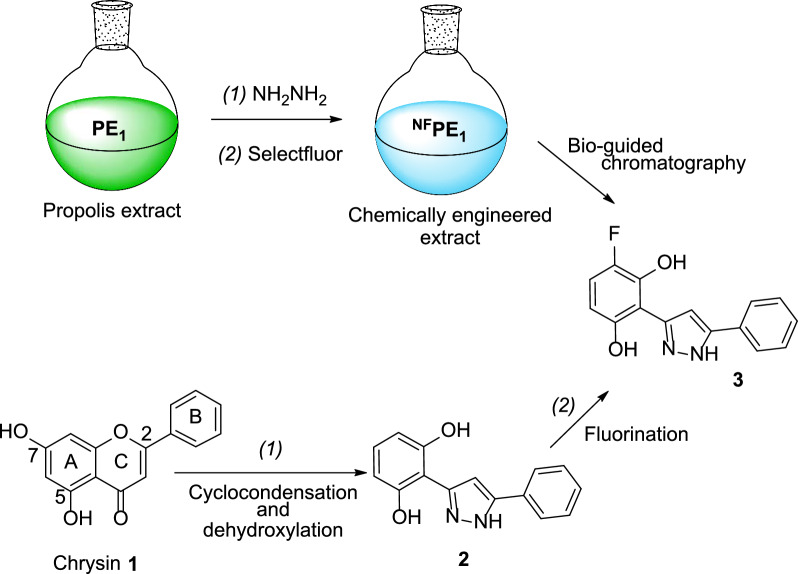
Reaction sequence that could lead to compound **3**

Fractionation of ^**NF**^**PE**_**1**_ using medium-pressure liquid chromatography (MPLC) bio-guided by the TLC α-Glc-inhibition assay led to the isolation of compound **3** (Scheme 1). This type of 3,5-diaryl pyrazoles can be produced by the reaction of hydrazine with flavones through a nucleophilic attack of hydrazine at the C-2 followed by ring-opening and further nucleophilic attack of the second nitrogen atom at the carbonyl carbon and subsequent dehydration [4, 11]. In addition, hydrazine monohydrate can lead to the selective dehydroxylation of aryl pyrazoles with phloroglucinol-type substitution pattern on the A-ring [48]. According to this mechanism, the natural flavone chrysin (**1**), if present in the precursor propolis extract **PE**_**1**_, could have reacted with hydrazine to produce compound **2**, which, upon fluorination, would lead to pyrazole **3** isolated from ^**NF**^**PE**_**1**_ (Scheme 1).

### Identification of the natural precursor of fluorinated pyrazol 3 and comparison of their inhibition properties

To corroborate the precursor-product relationship between chrysin (**1**) and pyrazole **3**, firstly, the presence of the natural flavone was confirmed in the propolis extract used as the starting material (**PE**_**1**_) by HPLC, TLC, and ^1^H NMR (Fig. S2).

**Fig. 3:**
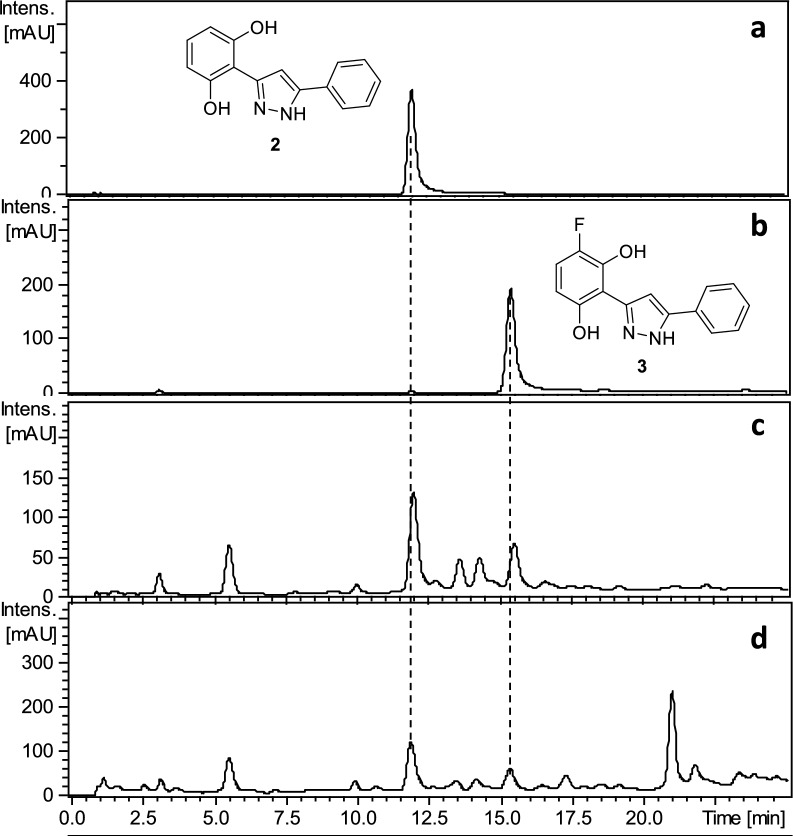
HPLC–UV-275 nm chromatograms of compound **2** (**a**), compound **3** (**b**), reaction mixture of the reaction with chrysin (**c**), and ^**NF**^**PE**_**1**_ (**d**)

Secondly, pure chrysin was treated sequentially with hydrazine and Selectfluor^®^ following the same procedure previously used for preparing ^**NF**^**PE**_**1**_ from **PE**_**1**_ (Scheme 1). HPLC analysis of the reaction mixture showed the formation of the two expected pyrazoles **2** and **3** (Fig. 3), which were later isolated by MPLC in 28% and 15% yield, respectively. This confirmed that chrysin was the natural precursor of **3** (Scheme1).

The α-Glc inhibition properties of chrysin and pyrazoles **1** and **2** were evaluated using a standard solution-based method. Chrysin inhibited α-Glc with an IC_50_ value of 212.30 ± 1.17 μM, which is more than 5 times higher than the value observed for the pyrazole **2** (IC_50_ = 38.27 ± 1.03 μM) and 20 times higher than the value observed for the fluorinated pyrazole **3** in the same experimental conditions (IC_50_ = 10.16 ± 1.23 μM). Furthermore, this fluorinated derivative (**3**) is twice more active than the reference α-Glc inhibitor acarbose (IC_50_ = 20.45 ± 1.02 μM). Physicochemical descriptors of compound **3** were computed to predict its drug-like properties using the SwissADME tool [49]. The compound demonstrated favorable drug-likeness, with no violations of the Lipinski [50], Ghose [51], Veber [52], Egan [53], or Muegge [54] filters. Additionally, no pan-assay interference structure (PAINS) alerts [55] or other structural alerts [56] were observed.

## Conclusions

Two sequential reactions were applied to introduce nitrogen and/or fluorine into the natural components of a series of EOs and PEs. In some cases, the procedure led to a positive change in the inhibitory properties of the enzyme α-Glc by the extracts. One of the chemically engineered extracts (^**NF**^**PE**_**1**_) showed interesting α-Glc inhibition properties due to the presence of a fluorinated pyrazole. This compound resulted from the reaction of hydrazine with the B-ring of chrysin, which is present in the starting propolis extract (**PE**_**1**_), leading to the formation of the pyrazole ring. Additionally, hydrazine reacted with the A-ring resulting in dehydroxylation at position 7. Further, selective fluorination of the A-ring led to the formation of pyrazole **3**, which inhibits α-Glc with a lower IC_50_ value than that of acarbose.

The results illustrate the potential of this strategy to generate and identify bioactive compounds. It is worth noting that in this particular case, the bioactive product is the result of the action of two reagents (hydrazine and Selectfluor^®^) on four common functional groups present in natural products (carbonyl, phenol, aromatic carbon, and a double bond), which led to the opening of a 6-member oxygenated heterocycle to produce a 5 member nitrogenated one, as well as the respective dehydroxylation and fluorination in two different positions of one of the aromatic rings of the natural starting material, all within a complex mixture of natural products. Overall, these transformations led to an approximately 20-fold increase in the α-Glc inhibition by both the whole compound mixture (4% inhibition by the starting natural **PE**_**1**_ *versus* 95% by the chemically engineered ^**NF**^**PE**_**1**_ at the same concentration) and the isolated inhibitor (IC_50_ = 10 μM for the semisynthetic compound **3** *versus* IC_50_ = 212 μM for its natural precursor chrysin **1**).

The process also highlights the utility of TLC-based inhibition assays to locate bioactive compounds within compound mixtures, by easily producing an α-Glc inhibition profile of the mixture before any purification step. This early information is useful as bioguide and facilitates the subsequent effect-directed purification of the corresponding inhibitor.

Overall, the approach suggests a shift in thinking: numerous semisynthetic compounds are generated simultaneously, and without knowing the exact outcome of the applied chemical process, bio-guided mixture selection and purification ensure that the most time-consuming work begins only after a promising compound has been detected.

## Experimental

### Preparation of CEEs

Since the exact composition of the natural mixtures used as starting material is unknown, some average properties of EOs or propolis components were used to estimate the appropriate amount of each reagent. The number of moles of reacting molecules was estimated considering 150 Da as the average molecular weight of EOs, 300 Da as the average molecular weight of propolis extract components, and one, as the average number of reacting group per molecule. Finally, 20 mol of hydrazine monohydrate and 1.1 mol of Selectfluor^®^ per “estimated” mol of starting mixture components were used for the reactions.

Typical procedure for preparation of ^NF^EOs: A solution of EO_1_ (100 mg, 0.66 mmol taking an average MW of 150 Da) and hydrazine monohydrate (645 μL, 13.32 mmol) in ethanol (5 mL) was stirred for 20 h under reflux. The reaction solution volume was reduced approximately to 1/3 under reduced pressure, water was added (5 mL), and the resulting solution was extracted with dichloromethane (DCM) (3 × 5 mL). The DCM fractions were dried (anh. Na_2_SO_4_), filtered, and evaporated at reduced pressure. The obtained crude (71.9 mg, 0.48 mmol) was dissolved in ethanol (5 mL), Selectfluor^®^ was added (186.8 mg, 0.53 mmol), and the solution was stirred at room temperature for 20 h. Water was added (5 mL) and the aqueous solution was extracted with DCM (2 × 5 mL). The DCM fractions were dried (anh. Na_2_SO_4_), filtered, and evaporated at reduced pressure.

Typical procedure for preparation of ^NF^PEs: A solution of PE_1_ (100 mg, 0.33 mmol taking an average MW of 300 Da) and hydrazine monohydrate (322 μL, 6.66 mmol) in ethanol (5 mL) was stirred for 20 h under reflux. The reaction solution volume was reduced approximately to 1/3 under reduced pressure, water was added (5 mL), and the resulting solution was extracted with DCM (3 × 5 mL). The DCM fractions were dried (anh. Na_2_SO_4_), filtered, and evaporated at reduced pressure. The obtained crude (51.5 mg, 0.17 mmol) was dissolved in ethanol (5 mL), Selectfluor® was added (66.90 mg, 0.19 mmol) and the solution was stirred at room temperature for 20 h. Water was added (5 mL) and the aqueous solution was extracted with DCM (2 × 5 mL). The DCM fractions were dried (anh. Na_2_SO_4_), filtered, and evaporated at reduced pressure.

### Fractionation of ^NF^PE_1_

The ^**NF**^**PE**_**1**_ was chromatographed in MPLC-UV (Elldex-Alltech). 50.0 mg were directly loaded on a Latek model M2 (2 cm Id × 33 cm length) glass column filled with Silica gel 60 RP-18 (15–25 μm, LiChroprep RP-18, Merck). Mobile phase: 1% Formic acid solution and methanol. Method: 0–60 min 60% methanol, 100 min 100% methanol. Injection solvent: methanol. Flow: 4 mL/min. The effluent of the column was monitored at 254 nm. Fractions were automatically collected every two minutes to obtain fifty fractions (F1 − F50). The fractions F13-15 contained 1.5 mg of pure of pure fluorinated pyrazole **3** (3.0% final yield).

### Synthesis of pyrazoles 2 and 3

Hydrazine monohydrate (390 µL, 7.8 mmol) was added to a solution of chrysin (100 mg, 0.393 mmol) in absolute ethanol (7 mL), and the mixture was stirred for 20 h under reflux. After that, the reaction solution volume was reduced approximately to 1/3 under reduced pressure, water was added (7 mL), and the resulting solution was extracted with DCM (3 × 7 mL). The DCM fractions were dried (anh. Na_2_SO_4_), filtered, and evaporated at reduced pressure. The obtained crude (87.0 mg, 0.34 mmoles) was dissolved in ethanol (7 mL), Selectfluor^®^ was added (132.49 mg, 0.37 mmol) and the solution was stirred at room temperature for 20 h. Water was added (7 mL) and the aqueous solution was extracted with DCM (2 × 7 mL). The DCM fractions were dried (anh. Na_2_SO_4_), filtered, and evaporated at reduced pressure. The mixture was purified by MPLC-UV on reversed-phase silica gel using methanol/H_2_O gradients to obtain pyrazole **2** (22.8 mg, 28.5% yield) and pyrazole **3** (12.2 mg, 15.2% yield).

Pyrazole **2**. Mp: 168–170 °C. ^1^H NMR (300 MHz, (CD_3_)_2_CO): δ = 7.83–7.79 (m, 2H, Ar–H); 7.49–7.44 (m, 3H, Ar–H and Pyr-H); 7.39–7.37 (m, 1H, Ar–H); 6.96 (t, 1H, *J* = 8.10 Hz, Ar–H); 6.46 (d, 2H, *J* = 8.10 Hz, Ar–H). ^13^C NMR (75 MHz, (CD_3_)_2_CO): δ C_1_ = C_3_ = 157.99; C_3’_ = 150.57; C_5’_ = 143.70; C_1’’_ = 130.45; C_3’’_ = C_5’’_ = 129.87; C_4’’_ = 129.31; C_5_ = 129.27; C_2’’_ = C_6’’_ = 126.35; C_4_ = C_6_ = 107.99; C_2_ = 106.10; C_4’_ = 104.76. IR (neat) ν = 3401, 1701, 1626, 1454, 1015, 768, 694 cm^−1^. HRMS: found m/z = 275.0783, calculated m/z for C_15_H_12_N_2_O_2_Na [M + Na]^+^ = 275.0791 (0.8 mDa error).

Pyrazole **3**. Mp: 133–134 °C. ^1^H NMR (300 MHz, (CD_3_)_2_CO: δ = 10.65 (1H, HO-Ar); 10.12 (1H, HO-Ar); 7.86 (m, 2H, Ar–H); 7.55 (s, 1H, Pyr-H); 7.52 (m, 2H, Ar–H); 7.43 (m, 1H, Ar–H); 6.94 (dd, 1H, *J*_*1*_ = 8.26 Hz,* J*_*2*_ = 4.08 Hz, Ar–H); 6.44 (dd, 1H, *J*_HH_ = 8.9 Hz, *J*_HF_ = 10.5 Hz, H-Ar). ^13^C NMR (75 MHz, (CD_3_)_2_CO: δ C_1_ = 153.51; C_3’_ = 150.40; C_4_ = 146.53 (d, ^1^J_C-F_ = 228.89 Hz); C_3_ = 145.40 (d, ^2^J_C-F_ = 15.46 Hz); C_5’_ = 144.00; C_1’’_ = 130.31; C_3’’_ = C_5’’_ = 130.10; C_4’’_ = 129.65; C_2’’_ = C_6’’_ = 126. 61; C_5_ = 115.35 (d, ^2^J_C-F_ = 19.66 Hz); C_2_ = 107.88 (d, ^3^J_C-F_ = 1.90 Hz); C_6_ = 106.48 (d, ^3^J_C-F_ = 6.75 Hz); C_4’_ = 104.92. ^19^F NMR (282 MHz, (CD_3_)_2_CO: δ = − 150.67 (^1^F, m). IR (neat) ν = 3415, 1703, 1634, 1494, 1462, 1026, 982, 854, 766, 695 cm^−1^. HRMS: found m/z = 271.0881, calculated m/z for C_15_H_11_FN_2_O_2_ [M + H]^+^ = 293.0877 (0.4 mDa error).

### Microplate α-glucosidase inhibition assays

The hydrolysis of *p*-nitrophenyl-α-O-D-glucopyranoside (α-pNPG) was continuously measured in a 96-well microplate using a method similar to that applied by Arnaldos et al. [57]. Wells were filled in triplicate with α-Glc (yeast) in 0.1 M, pH 7 phosphate buffer (0.088 U/mL end concentration per well), α-cyclodextrin, the same buffer solution (1.22 mM end concentration per well) and 10 µL of test compound in dimethylsulfoxide (DMSO) solution. Wells containing the corresponding volume of DMSO without an inhibitor were used as the references of maximum enzymatic rates. The final volume per well was 270 µL. The enzymatic reaction was initiated by adding α-pNPG (1.63 mM end concentration per well). The plate was shaken for 2 s and the increase in absorbance at 405 nm was monitored at 37 °C for 10 min.

DMSO solutions (0.084 mg/mL) of doubly chemically modified extracts were employed for α-Glc inhibition percentage determination.

For IC_50_ determination, ten serial dilutions of the compounds were prepared in DMSO, following equally spaced points on a neperian logarithm scale, starting at 64.7 mM and finishing at 0.00647 mM (end concentration per well: 2397 to 0.2514 µM). IC_50_ calculated using Prism V5.01 (GraphPad Software Inc., La Jolla, CA, USA) applying a non linear regression curve fit for a log[inhibitor] vs. normalized answer model with variable slope. Standard drug acarbose was used as enzyme inhibition control.

### TLC α-glucosidase inhibition assays

The α-Glc inhibition properties of the mixtures were surveyed by TLC autography using reported protocols [47]. Briefly, a Silica gel-TLC plate (64 cm^2^) was sprayed with the 2-naphthyl-α-d-glucopyranoside: Fast Blue B salt (1:1, V/V) solution using a glass reagent sprayer operated with compressed air. Then, the plate was dried under air current at room temperature. 80 mg of agar was dissolved at 80 °C in 9.4 mL phosphate buffer (100 mM, pH 7.0), the solution was allowed to cool down (40 °C) and 188 μL of α-Glc solution (12.5 U/mL) was added. The obtained solution was mixed by inversion and distributed over the TLC plate. After cooling and solidification, the plate was incubated at 37 °C (10 min) in a stove.

### HPLC conditions

DAD-HPLC measurements were performed on a Hewlett Packard HP 1050 series, coupled to a G1306AX DAD. The samples were directly loaded on a Phenomenex Gemini C_18_ column (5 µm × 150 mm × 4.6 mm). Mobile phase: A: Methanol with formic acid (5%); B water/methanol (39/61) with formic acid (5%). Method: 0 min, B 100%, 3 min B 100%, 5 min B 90%, 8 min B 90%, 15 min B 82%, 16 min B 82%, 17 min B 70%, 20 min B 60%, 22 min B 60%, 23 min B 10%, 25 min B 10%. Injected volume: 3 µl. Flow: 0.3 mL/min. Column temperature: 30.0 °C. Detection at 254 nm. Solutions of 0.15 mg/mL (pure compounds) or 10 mg/mL (for mixture) were injected.

## Supplementary Information

Additional file 1. Chemical reagents, materials, and equipment details, Table S1, Figures S1 and S2, NMR and HRMS spectra of compounds 2 and 3.

## Data Availability

The data supporting the results of this study can be obtained from the corresponding authors upon reasonable request.
