# Supplementary material for: New semisynthetic α-glucosidase inhibitor from a doubly-chemically engineered extract
Source: Nat Prod Bioprospect. 2025 Jan 5;15(1):4. doi: 10.1007/s13659-024-00488-2 (PMC11700072; doi:10.1007/s13659-024-00488-2)
Supplement: Supplementary file 1 — Additional file 1. Chemical reagents, materials, and equipment details, Table S1, Figures S1 and S2, NMR and HRMS spectra of compounds 2 and 3. [file 13659_2024_488_MOESM1_ESM.docx]

**Supplementary Information for:**

**New semisynthetic α-Glucosidase Inhibitor from a Doubly-Chemically Engineered Extract**

María I. Osella,^[b] ‡^ Mario O. Salazar,^[a, b] ‡^ Carlos M. Solís, ^[a]^ and Ricardo L. E. Furlan*^[a, b]^

[a] Dr. Mario O. Salazar, Dr. Carlos M. Solís, Dr. Ricardo. L. E. Furlan
Farmacognosia, Facultad de Ciencias Bioquímicas y Farmacéuticas
Universidad Nacional de Rosario
Suipacha 531, S2002LRK Rosario (Argentina)
E-mail: rfurlan@fbioyf.unr.edu.ar

[b] Dr. Maria I. Osella, Dr. Mario O. Salazar, Dr. Ricardo. L. E. Furlan
Consejo Nacional de Investigaciones Científicas y Técnicas
Suipacha 531, S2002LRK Rosario (Argentina)

* Corresponding author.

E-mail address: [rfurlan@fbioyf.unr.edu.ar](mailto:rfurlan@fbioyf.unr.edu.ar) (R. L. E. Furlan). Phone: +54-0341-4375315. https://orcid.org/0000-0001-6136-0980

‡These authors contributed equally.

**Contents pagina**

**Chemical reagents, enzymes, and equipments S2**

**Table S1 S2**

**Figure S1 S3**

**Figure S2 S3**

**^1^H NMR of compound 2 S4**

**^13^C NMR of compound 2 S4**

**HRMS of compound 2 S4**

**^1^H NMR of compound 3 S5**

**^13^C NMR of compound 3 S5**

**^19^F NMR of compound 3 S6**

**HRMS of compound 3 S6**

**Chemical reagents, enzymes and equipment**

Chemical reagents and enzymes were purchased from commercial sources and were used without further purification. Solvents were analytical grade or were purified by standard procedures prior to use. Aluminum-blacked silica gel 60 F and silica gel 60 RP-18 F254 TLC layers, and LiChroprep 254 RP-18 were purchased from Merck (Darmstadt, Germany). Agar was purchased from Britania (Buenos Aires, Argentina). Sodium sulfate, hydrazinemonohydrate, Selectfluor^®^, *α*-Glucosidase (*α*-glc, from *Saccharomyces cerevisiae*, EC 3.2.1.20), Fast Blue B salt (FBB), Tris(hydroxymethyl)aminomethane (Tris), *p*-nitrophenyl-α-O-D-glucopyranoside, α-cyclodextrin, 2-aminoethyl diphenylborate (NP), poly(ethylene glycol) 4000 (PEG) and crysin were purchased from Sigma-Aldrich (St. Louis, MO, USA). Sodium phosphate dibasic and sodium phosphate monobasic was purchased in Cicarelli (San Lorenzo, Argentina).The 2-Naphthyl-*α*-d-glucopyranoside (*α*-Nglc) was acquired from Glycosynth (Warrington, Cheshire, England). Acarbose (produced by Cayman Chemical, Cambrige, United Kindong) was acquired from Migliorelaclaustra, Argentine.

The essential oils (EOs) were purchased from EUMA (Buenos Aires, Argentina): *Salvia officinalis* L. , *Juniperus communis* L., *Juniperus virginiana* L., *Cinnamomum cassia* (Nees & T.Nees) J.Presl, *Thuya occidentalis* L., *Litsea cubeba* (Lour.) Pers., *Cymbopogon citratus* Spreng, *Artemisia absinthium* L., *Eugenia caryophyllata* (L.) Merr. and L.M.Perry, *Cananga odorata* (Lam.) Hook.f. & Thomson. The propoleis samples were was donated by differents Argentine producers: Esperanza and Reconquista, Santa Fe province, Lucas González, Entre Ríos province, and Dean Funes Córdoba province.

^1^H NMR spectra were recorded on a Bruker avance II at 300 MHz in (CD_3_)_2_CO, in the presence of TMS (0.00 ppm) as the internal standard. ^13^C NMR spectra were recorded on the same apparatus at 75 MHz in (CD_3_)_2_CO, in the presence of TMS (0.00 ppm) as the internal standard. ^19^F NMR spectra were recorded on the same apparatus at 282 MHz in (CD_3_)_2_CO. High Resolution Mass spectra (direct infusion MS experiments) were recorded on a Q-Exactive (Thermo Fisher Scientific). Methanol (acquired from Carlo Erba) was used for samples preparation. MS: source type, ESI; ion polarity, positive; set nebuliser, 0.4 Bar; set dry heater, 180°C; set dry gas, 4.0 L/min; set capillary, 4500 V; set end plate offset, 500 V; set collision cell radio frequency, 150.0 Vpp. ISC.

**Table S1:** The mass recovery for **EOs** and **PEs** for the reactions with hydrazine (first reaction) and Selectfluor (second reaction).

|  |  | Recovered mass (%) | |  |
| --- | --- | --- | --- | --- |
| Sample | Initial mass (mg) | Reacc. with hydrazine | Reacc. with Selectfluor | Total mass (mg) |
| EO_1_ | 98.1 | 71.9 | 76.4 | 53.9 |
| EO_2_ | 99.4 | 72.8 | 66.2 | 47.9 |
| EO_3_ | 104.9 | 104.9 | 75.9 | 83.5 |
| EO_4_ | 99.1 | 89.4 | 86.3 | 76.5 |
| EO_5_ | 98.8 | 79.8 | 73.2 | 57.7 |
| EO_6_ | 95.7 | 89.4 | 85.4 | 73.1 |
| EO_7_ | 97.8 | 91.1 | 97.2 | 86.6 |
| EO_8_ | 98.3 | 85.2 | 70.1 | 58.7 |
| EO_9_ | 100.9 | 99.2 | 89.2 | 89.3 |
| EO_10_ | 97.0 | 93.6 | 81 | 73.5 |
| PE_1_ | 121.2 | 51.5 | 89.5 | 55.9 |
| PE_2_ | 103.3 | 78.3 | 83.1 | 67.2 |
| PE_3_ | 86.3 | 82.8 | 90.1 | 64.4 |
| PE_4_ | 117.2 | 56.7 | 74.9 | 49.8 |

**Fig. S1** α-Glc inhibition at 12.5, 3.125, and 0.781 µg/mL by ^NF^EOs and ^NF^PEs: ^NF^EO_1_ (*S officinalis*), ^NF^EO_2_ (*J.* *communis*), ^NF^EO_3_ (*J.* *virginiana*), ^NF^EO_4_ (*C.* *cassia*), ^NF^EO_5_ (*T. occidentalis*), ^NF^EO_6_ (*L. cubeba*), ^NF^EO_7_ (*C.* citratus), ^NF^EO_8_ (*A. absinthium*), ^NF^EO_9_ (*E. caryophyllata*), ^NF^EO_10_(*C. odorata*), ^NF^PE_1_(Esperanza, Santa Fe province), ^NF^PE_2_ (Reconquista, Santa Fe province, PE2), ^NF^PE_3_ (Lucas González, Entre Ríos province), and ^NF^PE_4_ (Dean Funes Córdoba province)

**Fig. S2** a**)** HPLC-UV-275 nm chromatograms of **1** (up) and **PE_1_** (down), **b)** TLC of **1** (left) and **PE_1_** (right) under UV lights 254 nm, 365 nm, visible NP-PEG, and NP-PEG/365 nm. Mobile phase: hexane-ethyl acetate (1:1), and **c)** ^1^H NMR spectras of **1** (up) and **PE_1_** (down)

^1^H NMR of pyrazole **2**, 300 MHz, CO(CD_3_)_2_

^13^C NMR of pyrazole **2**, 75 MHz, CO(CD_3_)_2_

HRMS of pyrazole **2**

^1^H NMR of pyrazole **3**, 300 MHz, CO(CD_3_)_2_

^13^C NMR of pyrazole **3**, 75 MHz, CO(CD_3_)_2_

^19^F NMR of pyrazole **3**, 282 MHz, CO(CD_3_)_2_

HRMS of pyrazole **3**
